# Risk of arbovirus emergence via bridge vectors: case study of the sylvatic mosquito *Aedes malayensis* in the Nakai district, Laos

**DOI:** 10.1038/s41598-020-64696-9

**Published:** 2020-05-08

**Authors:** Elliott F. Miot, Elodie Calvez, Fabien Aubry, Stéphanie Dabo, Marc Grandadam, Sébastien Marcombe, Catherine Oke, James G. Logan, Paul T. Brey, Louis Lambrechts

**Affiliations:** 1 Insect-Virus Interactions Unit, Institut Pasteur, UMR2000, CNRS, Paris, France; 2 Sorbonne Université, Collège doctoral, Paris, France; 3 Medical Entomology and Vector-Borne Disease Unit, Institut Pasteur du Laos, Vientiane, Lao PDR; 4 Arbovirus and Emerging Viral diseases Laboratory, Institut Pasteur du Laos, Vientiane, Lao PDR; 5 Faculty of Infectious and Tropical Diseases, London School of Hygiene and Tropical Medicine, London, United Kingdom

**Keywords:** Ecological epidemiology, Viral infection

## Abstract

Many emerging arboviruses of global public health importance, such as dengue virus (DENV) and yellow fever virus (YFV), originated in sylvatic transmission cycles involving wild animals and forest-dwelling mosquitoes. Arbovirus emergence in the human population typically results from spillover transmission via bridge vectors, which are competent mosquitoes feeding on both humans and wild animals. Another related, but less studied concern, is the risk of ‘spillback’ transmission from humans into novel sylvatic cycles. We colonized a sylvatic population of *Aedes malayensis* from a forested area of the Nakai district in Laos to evaluate its potential as an arbovirus bridge vector. We found that this *Ae. malayensis* population was overall less competent for DENV and YFV than an urban population of *Aedes aegypti*. Olfactometer experiments showed that our *Ae. malayensis* colony did not display any detectable attraction to human scent in laboratory conditions. The relatively modest vector competence for DENV and YFV, combined with a lack of detectable attraction to human odor, indicate a low potential for this sylvatic *Ae. malayensis* population to act as an arbovirus bridge vector. However, we caution that opportunistic blood feeding on humans by sylvatic *Ae. malayensis* may occasionally contribute to bridge sylvatic and human transmission cycles.

## Introduction

Many emerging arthropod-borne viruses (arboviruses) of humans such as dengue, Zika, yellow fever and chikungunya viruses originated in sylvatic transmission cycles where they circulate between vertebrate animals and forest-dwelling mosquitoes^1^. Over the last few centuries, these arboviruses have emerged into sustained transmission cycles among humans, causing substantial mortality and morbidity. Zoonotic arboviruses are typically brought into the human population by mosquitoes referred to as bridge vectors, which make the link between sylvatic and human transmission cycles. Bridge vectors are competent for arbovirus transmission and display host-feeding behavior towards both humans and non-human animals. The emergence or re-emergence of sylvatic arboviruses into the human population is an important public health concern^2^ that calls for careful investigations at the interface between human populations and the sylvatic environment.

Another related, but less studied concern, is the risk of ‘spillback’ of arboviruses from the human population into novel sylvatic cycles in regions where they were previously absent^3^. Not only are newly established sylvatic arbovirus cycles virtually impossible to eradicate, but such spillback events can have potentially serious consequences for wildlife. This was exemplified by yellow fever virus (YFV), which was introduced into the Americas from West Africa about 400 years ago during the slave trade and contributed to shape the expansion of settlements and colonial powers^4^. YFV also established sylvatic transmission cycles within the Amazon, Araguaia, and Orinoco river basins where it has been responsible for deadly outbreaks among non-human primates of the New World. The incriminated bridge vectors were mosquitoes in the genera *Haemagogus* and *Sabethes*^5^. Recent reports from Kenya suggest that human dengue virus (DENV) infections may be a source of ‘spillback’ transmission to baboon populations^6^.

The Nakai National Biodiversity Conservation Area, Nakai district, Khammouane province consists of dry evergreen forests, cloud forests and mountainous riverbeds that are the biotopes of various mosquito species and monkeys with increasing incursions of humans. About 10,000 humans (1.95 persons/km^2^) reside in and around this area bordering and within the primary forest of the Annamite Range^7^. The mosquito *Aedes malayensis* in the *Stegomyia* sub-genus is widely distributed in South-East Asia with records in Thailand, Cambodia, Vietnam, Peninsular Malaysia, the Andaman and Nicobar Islands, and Taiwan^8–10^. It was also identified in peridomestic habitats of Singapore as a putative vector of YFV, DENV and chikungunya virus^11,12^. Recently, our mosquito surveys in the Nakai National Biodiversity Conservation Area indicated the presence of *Ae. malayensis* in this area^13,14^.

Here, we evaluated the potential of *Ae. malayensis* to act as an arbovirus bridge vector in the Nakai National Biodiversity Conservation Area, using a combination of vector competence assays and behavioral experiments. Relative to *Ae. aegypti* controls, we found that our field-derived *Ae. malayensis* colony had similar vector competence indices for DENV type 1, but a lower susceptibility to YFV. In addition, olfactometer bioassay measurements showed that this *Ae. malayensis* population was not significantly attracted to human odor in laboratory conditions. We conclude that although this sylvatic *Ae. malayensis* population does not display a strong attraction to human odor, its vector competence for arboviruses such as DENV may contribute to bridge sylvatic and human transmission cycles when it engages in opportunistic blood feeding on humans.

## Results

### Lower DENV-1 vector competence of *Ae. malayensis* relative to *Ae. aegypti*

**Figure 1:**
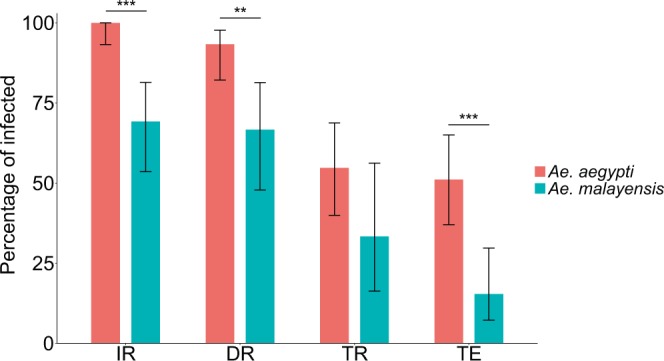
Vector competence of sylvatic *Ae. malayensis* and *Ae. aegypti* controls after exposure to 1.16–1.38 ×10^7^ FFUs/ml of DENV-1. Bars represent the percentage of virus-positive mosquitoes 14 days post infectious blood meal and the error bars are the 95% confidence intervals of the percentages. Infection rate (IR) is the proportion of blood-fed females with an infected body. Dissemination rate (DR) is the proportion of infected females with virus disseminated to the head tissues. Transmission rate (TR) is the proportion of females with a disseminated infection that shed virus in their saliva. Transmission efficiency (TE) is the overall proportion of blood-fed females that shed virus in their saliva. The *Ae. aegypti* population was included as a positive control. The figure compiles data from two independent experiments that did not differ significantly. Blood meal titers were 1.16 ×10^7^ and 1.38 ×10^7^ FFUs/ml in the first and second experiment, respectively. **p < 0.01; ***p < 0.001.

We measured the DENV-1 vector competence of 39 *Ae. malayensis* females and 53 *Ae. aegypti* females in two separate experiments. The data from both experiments were combined because initial analyses showed that none of the vector competence indices differed significantly between them. In each experiment, mosquitoes were exposed to an infectious dose of 1.16–1.38 ×10^7^ FFU/ml of DENV-1. Vector competence was analyzed 14 days post infectious blood meal. The proportion of blood-fed females that became infected (i.e., the infection rate; IR) was 69.2% (27/39) and 100% (53/53) for *Ae. malayensis* and *Ae. aegypti*, respectively (Fig. 1). The difference in IR between the two species was statistically significant (*p* < 0.0001). The proportion of infected females that developed a disseminated infection (i.e., the dissemination rate; DR) was 66.7% (18/27) for *Ae. malayensis* and 93.3% (42/45) for *Ae. aegypti* (Fig. 1). The difference in DR between the two species was statistically significant (*p* = 0.004). The proportion of females with a disseminated infection that also released virus in their saliva (i.e., the transmission rate; TR) was 33.3% (6/18) for *Ae. malayensis* and 54.8% (23/42) for *Ae. aegypti* (Fig. 1). The difference in TR between the two species was not statistically significant (*p* = 0.125). The overall proportion of blood-fed females with virus-positive saliva (i.e., the transmission efficiency; TE) was 15.4% (6/39) and 51.1% (23/45) for *Ae. malayensis* and *Ae. aegypti*, respectively (Fig. 1). The difference in TE between the two species, which summarizes their relative vector competence, was statistically significant (*p* = 0.0004). Together, these data indicate that *Ae. malayensis* is a competent DENV-1 vector, but to a lesser extent than the *Ae. aegypti* control population.

### **Lower YFV vector competence of*Ae. malayensis*relative to*Ae. aegypti***

**Figure 2:**
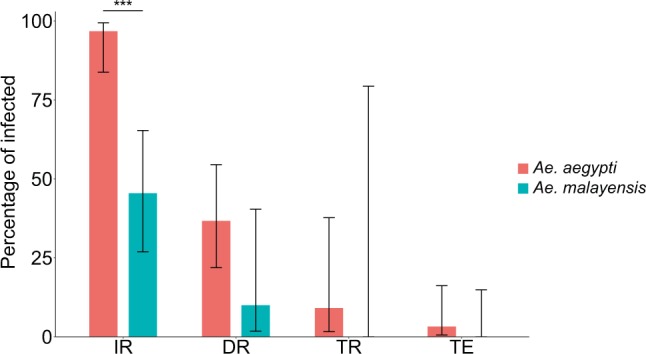
Vector competence of sylvatic *Ae. malayensis* and *Ae. aegypti* controls after exposure to 1.84 ×10^6^ FFUs/ml of YFV. Bars represent the percentage of virus-positive mosquitoes 14 days post infectious blood meal and the error bars are the 95% confidence intervals of the percentages. Infection rate (IR) is the proportion of blood-fed females with an infected body. Dissemination rate (DR) is the proportion of infected females with virus disseminated to the head tissues. Transmission rate (TR) is the proportion of females with a disseminated infection that shed virus in their saliva. Transmission efficiency (TE) is the overall proportion of blood-fed females that shed virus in their saliva. The *Ae. aegypti* population was included as a positive control. ***p < 0.001.

We analyzed the YFV vector competence of 22 *Ae. malayensis* and 31 *Ae. aegypti* 14 days after oral exposure to an infectious blood meal containing 1.84 ×10^6^ FFU/ml of YFV. The IR was 45.5% (10/22) and 96.8% (30/31) for *Ae. malayensis* and *Ae. aegypti*, respectively (Fig. 2) and the difference between the two species was statistically significant (*p* < 0.001). The DR was 10% (1/10) and 36.7% (11/30) for *Ae. malayensis* and *Ae. aegypti*, respectively (Fig. 2), and the difference between the two species was not statistically significant (*p* = 0.087). The TR was 0% (0/1) and 9.1% (1/11) for *Ae. malayensis* and *Ae. aegypti*, respectively (Fig. 2), and the difference between the two species could not be statistically tested because there was only 1 *Ae. malayensis* female and thus no replication. Overall, there was no evidence for a statistically significant difference (*p* = 0.297) in vector competence between the two species, as TE was 0% (0/22) and 3.2% (1/31) for *Ae. malayensis* and *Ae. aegypti*, respectively (Fig. 2). However, the very small proportion of positive saliva samples overall (a single *Ae. aegypti* saliva sample was found positive) limited our ability to detect differences. Together, these data do not conclusively demonstrate the YFV vector competence of *Ae. malayensis*, but suggest that it is likely lower than that of the *Ae. aegypti* control population.

### **Lack of evidence for*Ae. malayensis*attraction to human odor**

**Figure 3:**
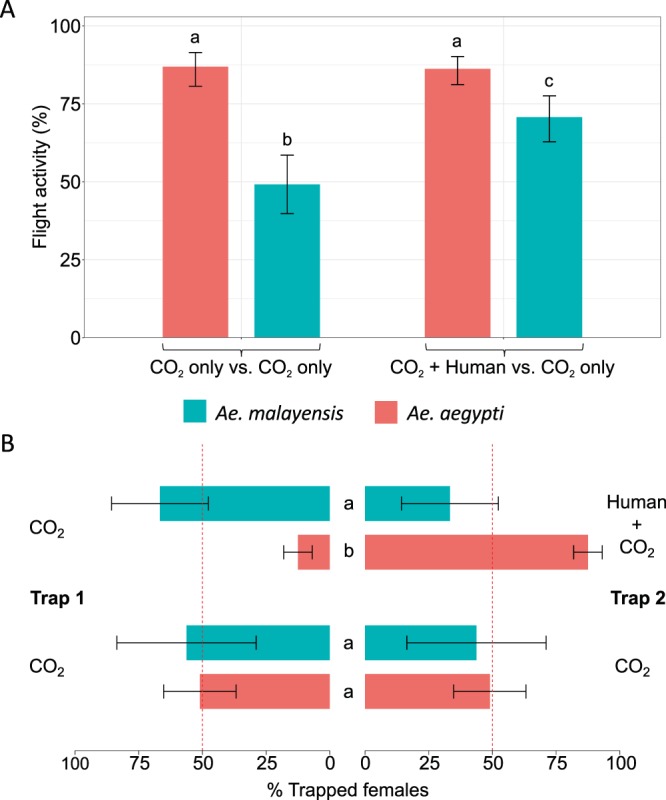
Lack of laboratory evidence for *Ae. malayensis* attraction to human odor. (**A**) Flight activity is the percentage of female mosquitoes that exited the release chamber after 20 min. (**B**) Attraction to human odor was estimated as the percentage of trapped mosquitoes that chose the trap with human odor, which ranges from 0% (full attraction to CO_2_ without human odor) to 100% (full attraction to CO_2_ with human odor). The red, vertical dashed lines indicate the expected percentage of trapped mosquitoes when there is no preference for either trap (50%). Error bars represent 95% confidence intervals of the percentages. Letters next to or above the bars indicate statistical significance. Conditions with a letter in common are not significantly different from each other. The data shown in (**A**) and (**B**) are pooled from 6 and 9 separate replicate trials of 20 to 30 females each for CO_2_ only vs. CO_2_ only and CO_2_ + Human odor vs. CO_2_ only designs, respectively, which did not differ significantly. The *Ae. aegypti* population was included as a positive control.

In addition to vector competence, the ability of *Ae. malayensis* to act as an arbovirus bridge vector depends on its human-biting behavior. A typical bridge vector is expected to display an intermediate level of attraction to humans, so that it bites humans and non-human animals in alternation. To assess its probability of human biting, we measured the attraction of *Ae. malayensis* to human odor using a dual-port olfactometer. Flight activity in the presence of human scent (i.e., in the CO_2_ + Human odor vs. CO_2_ only design) was significantly lower (*p* = 0.001) for *Ae. malayensis* than for *Ae. aegypti* with 70.7% (111/157) and 86.2% (213/247) of females exiting the release chamber after 20 min, respectively (Fig. 3A). However, the flight activity of *Ae. aegypti* was not significantly influenced (*p* = 0.997) by human odor as 86.9% (146/168) of females exited the release chamber in the CO_2_ only vs. CO_2_ only design (Fig. 3A), whereas the flight activity of *Ae. malayensis* was significantly enhanced (*p* = 0.002) in the presence of human odor with 49.1% (57/116) exiting the release chamber in the CO_2_ only vs. CO_2_ only design (Fig. 3A).

Attraction towards human odor of each species was estimated as a percentage of trapped females that were caught in the trap with human odor. This index of attraction varies from 0% (full attraction to CO_2_ without human odor) to 100% (full attraction to CO_2_ with human odor), with 50% indicating a neutral behavior (no effect of the human odor). To rule out any bias in the intrinsic attractiveness of the traps, we also performed control CO_2_ only vs. CO_2_ only experiments for each species. In the control experiments, 51% (95% CI: 36.8–65.2) of *Ae. aegypti* females and 56.2% (95% CI: 28.9–83.6) of *Ae. malayensis* females chose one trap over the other. For both species, the percentage of mosquitoes in each trap did not significantly differ from 50%, which confirmed the lack of bias between the traps. As expected, 87.5% (95% CI: 81.9–93.1) of trapped *Ae. aegypti* females chose the trap with human odor (Fig. 3B), indicating significant attraction to human odor. In contrast, 33.3% (95% CI: 14.3–52.3) of trapped *Ae. malayensis* chose the trap with human odor (Fig. 3B), and this percentage was not statistically different from 50% indicating a lack of response to human odor. Accordingly, relative attraction towards human odor was significantly lower for *Ae. malayensis* than for *Ae. aegypti* (*p* < 0.0001; Fig. 3B). Overall, these experiments revealed that *Ae. malayensis* was not attracted to human odor (i.e., it did not specifically choose the trap containing the human odor), but was activated by human odor (i.e., it initiated flight and host-seeking behavior) as more females responded to the combination of CO_2_ and human odor than to CO_2_ alone.

## Discussion

In this study, we demonstrated that a sylvatic *Ae. malayensis* population from a forested area of the Nakai district, Laos was competent for DENV-1 transmission, but to a lower extent than the *Ae. aegypti* control population. We also showed that this *Ae. malayensis* population had lower YFV vector competence indices than the *Ae. aegypti* control population, although TR was low for both species in our experimental conditions. Finally, whilst our olfactometer experiment did find greater flight activation by CO_2_ with human odor than by CO_2_ only, *Ae. malayensis* did not display attraction to human odor under these laboratory conditions. Overall, our assessment indicates a low potential for this sylvatic *Ae. malayensis* population to act as an arbovirus bridge vector in rural Laos.

Mosquito vector competence for arboviruses depends on several tissue barriers associated with the midgut and the salivary glands^15^. The physiological and molecular nature of these barriers is poorly understood, but they are used as conventional phenotypes to characterize vector competence. IR informs on the midgut infection barrier (i.e., preventing infection of midgut epithelium), DR represents the midgut escape barrier (i.e., preventing virus exit from the midgut cells into the haemocoel), and TR relates to both salivary glands infection and escape barriers^16^. Our sylvatic colony of *Ae. malayensis* was experimentally competent for DENV-1, but its overall TE ( = IR x DR x TR) was lower than that of the *Ae. aegypti* control. This difference resulted from significantly lower IR and lower DR, but not lower TR (Fig. 1). This pattern suggests that the lower vector competence of this *Ae. malayensis* population compared to the *Ae. aegypti* control is not primarily due to salivary glands barriers, but rather to midgut-related mechanisms hindering virus infection and dissemination. The pattern was different for YFV, for which all vector competence phenotypes were similar between the two species with the exception of IR (Fig. 2). In our experiment, the same oral infectious dose of YFV infected about twice less *Ae. malayensis* than *Ae. aegypti*, whereas subsequently DR and TR did not differ significantly. Nevertheless, the low TR overall limited our ability to detect a difference between the two species. Further studies will be necessary to elucidate the mechanisms underlying differences in vector competence between *Ae. malayensis* and *Ae. aegypti*.

The results obtained with this sylvatic population of *Ae. malayensis* in Laos should not be extrapolated to other populations of the species. Indeed, vector competence of a mosquito species for an arbovirus depends on the specific combination of the mosquito and the virus genotypes^17,18^ as well as on environmental factors such as ambient temperature^19^. The mosquito *Ae. malayensis* is widely distributed across South-East Asia with documented occurrence in Vietnam, Cambodia, Peninsular Malaysia and Singapore, Taiwan, the Andaman and Nicobar Islands, Laos, and Thailand^8–11,13^. Recently, a peridomestic population of *Ae. malayensis* in Singapore was shown to be competent for diverse arboviruses such as DENV, YFV and chikungunya virus^11,12^.

A human-baited trap survey demonstrated that this peridomestic *Ae. malayensis* in Singapore displayed an anthropophilic behavior^12^, as was also documented for other field populations of *Ae. malayensis* in South-East Asia^8,9^. The discrepancy between the present and the earlier studies could be due to several, non-mutually exclusive explanations. First, there could be biological differences in human-biting behavior between different populations of the same species, as was observed for *Ae. aegypti*^20,21^. Second, behavioral assays in the laboratory may lack critical cues underlying host-seeking behavior in the field. Indeed, multiple sensory modalities such as CO_2_, heat and visual cues that were not present in our simplified laboratory approach may contribute to attract *Ae. malayensis* to feed on humans in a natural situation^22,23^. Moreover, volatiles were collected from the foot only and *Ae. malayensis* might not respond to foot odor since metabolites and volatiles largely vary according to the human body part^24^. Additionally, the human-associated microbial flora may have been largely eliminated during −20 °C storage of the stocking used to collect human odor. Although our behavioral bioassay successfully detected a strong attraction of *Ae. aegypti* to human scent, it is possible that for *Ae. malayensis* the experimental setup did not faithfully represent a natural situation. Additionally, the fact that more *Ae. malayensis* responded to the combination of CO_2_ and human odor than CO_2_ only indicated that they were activated by human odor, implying that human odor was enough to initiate flight and stimulate the beginning of a host location response, but perhaps not follow through to closer-range behavior. It would be interesting to increase the complexity of the sensory signals available for the mosquitoes to better mimic the natural conditions, for example using a live host olfactometer assay^20^.

Arbovirus bridge vectors represent the most likely mechanism of arbovirus spillover transmission to humans. Whereas sylvatic YFV transmission is only known to occur in Africa and South America, sylvatic DENV transmission cycles have been documented in the forests of South-East Asia. In particular, sylvatic DENV transmission was reported in peninsular Malaysia where sylvatic DENV strains were isolated from sentinel monkeys such as the crab-eating macaque (*Macaca fascicularis*) in remote forest reserves^1,2,25–27^. Crab-eating macaques are distributed across South-East Asia and can also be found in the forests of southern Laos^28^. Whether sylvatic DENV strains circulate in forests of Laos in unknown, but their emergence in the human population is a concern in those areas^1^ because they can potentially result in severe disease^2^. The NNT NPA harbors abundant biodiversity with the presence of at least nine non-human primate (NHP) species such as macaques, gibbons, langurs, and red-shanked doucs^29^. Further investigations at the human-forest interface in Laos are necessary to evaluate the risk of spillover DENV transmission to humans.

Dengue is endemic in Laos with the circulation of at least three serotypes (i.e., DENV-1, -2 and -4) all year long and yearly epidemics during the rainy season^30,31^. Dengue epidemics occur not only in urban areas, but also in more rural settings. For instance, an outbreak of DENV-1 was detected in 2008 in a remote village of northwestern Laos^32^. DENV-3 was isolated from *Armigeres subalbatus* collected on the Nakai plateau^33^. DENV circulation in rural Laos increases the risk of spillback transmission to NHPs because most of the people living in rural Laos still depend on the forest to sustain themselves. Moreover, one of the goals of WMPA is to develop ecotourism in the NNT NPA. Similar projects exist in the country or are in development. Therefore, infected travelers represent another possible source of DENV introduction or re-introduction into the sylvatic environment. This risk is likely under-estimated as humans can transmit DENV to mosquitoes in the absence of clinical symptoms^34^.

To more fully assess the potential of *Ae. malayensis* as an arbovirus bridge vector, future studies will need to consider additional populations of *Ae. malayensis* found in various ecological habitats both in Laos and in surrounding countries of South-East Asia, as well as other virus strains and serotypes including sylvatic DENV isolates. In addition, it will be important to account for other parameters underlying the vectorial capacity such as the longevity of female mosquitoes.

## Conclusions

The relatively modest vector competence for DENV and YFV, combined with a lack of detectable attraction to human odor, indicate a low potential for this sylvatic *Ae. malayensis* population to act as an arbovirus bridge vector. However, we caution that opportunistic blood feeding on humans by sylvatic *Ae. malayensis* may occasionally contribute to bridge sylvatic and human transmission cycles. The presence of susceptible NHPs combined with the possibility of a human-mediated introduction of DENV into the sylvatic environment are additional risk factors that should be taken into account. Once entrenched, a newly established sylvatic cycle would undermine long-term DENV control in Laos.

## Methods

### Ethics approval and consent to participate

This study used human blood samples to prepare mosquito artificial infectious blood meals. Methods were carried out in accordance with relevant guidelines and regulations, and experimental protocols were approved by the French Ethical Committee Ile-de-France I. Healthy donor recruitment was organized by the local investigator assessment using medical history, laboratory results and clinical examinations. Biological samples were supplied through participation of healthy volunteers at the ICAReB biobanking platform (BB-0033-00062/ICAReB platform/Institut Pasteur, Paris/BBMRI AO203/[BIORESOURCE]) of the Institut Pasteur to the CoSImmGen and Diagmicoll protocols, which have been approved by the French Ethical Committee Ile-de-France I. The Diagmicoll protocol was declared to the French Research Ministry under reference DC 2008-68 COL 1. All adult subjects provided written informed consent.

### Mosquitoes

Experiments were carried out with a laboratory colony derived from a sylvatic population of *Ae. malayensis* (7^th^ generation) collected in March 2017 and subsequently maintained at the Institut Pasteur du Laos. The *Ae. malayensis* colony was initiated with mosquito larvae collected along the Nam Noy River (17.768548°N, 105.381989°E) in the Nakai Nam Theun National Protected Area (NNT NPA), known as the Watershed Management and Protection Authority (WMPA), located in the Nakai district, Khammuane province, Laos^13^. A laboratory colony of *Ae. aegypti* (7^th^ generation) maintained at the Institut Pasteur du Laos was used as a control. The *Ae. aegypti* colony originated in the town of Paksan (18.37134°N, 103.66586°E), Paksan district, Bolikhamxay province, Laos. Mosquitoes were reared under controlled insectary conditions (28 °C, 70% relative humidity, 12:12 hour light cycle) as previously described^12^. Eggs were hatched synchronously in a vacuum chamber for 1 hour. Larvae were reared in 24 ×34 ×9 cm plastic trays containing 1.5 L of dechlorinated tap water and fed with Tetramin (Tetra) fish food at a density of 400 larvae per tray. Eight hundred adults were kept in 30 ×30 ×30 cm Bugdorm-1 insect cages with permanent access to 10% sucrose solution. For olfactometer studies at the London School of Hygiene and Tropical Medicine, eggs from the *Ae. malayensis* and *Ae. aegypti* colonies were shipped to London and reared under similar insectary conditions.

### Viruses

Vector competence experiments were carried out following previously described methods^12^ with low-passage DENV type 1 (DENV-1) and YFV isolates. The DENV-1 isolate (H15–3000) was originally obtained in 2015 through the Institut Pasteur du Laos DENV surveillance network in Vientiane capital, and was passaged three times in *Aedes albopictus* C6/36 cells before it was used in this study. Virus stocks were produced during the third passage following as previously described^35^. The YFV isolate (YFV-S79 strain) belongs to the West African lineage and was originally obtained in 1979 from the serum of a patient returning to France from Senegal^36^. Prior passage history of the YFV isolate included two passages in newborn mouse brains and two passages in C6/36 cells, which occurred before the isolate was obtained for this study. Virus stocks were produced during an additional C6/36 passage, as previously described for DENV^35^. Virus titration was performed by standard focus-forming assay (FFA) as previously described for DENV^35^ and YFV^12^. A commercial mouse anti-dengue virus complex monoclonal antibody (MAB8705; Merck Millipore) diluted 1:200 in phosphate-buffered saline (PBS) supplemented with 1% bovine serum albumin (BSA; Interchim) was used as primary antibody for DENV. A commercial mouse anti-flavivirus group antigen monoclonal antibody (MAB10216; Merck Millipore) diluted 1:1,000 in PBS supplemented with 1% BSA (Interchim) was used as primary antibody for YFV.

### Oral challenge

Mosquitoes were orally challenged with DENV-1 in two separate experiments and with YFV in a third one, following previously described methods^12,31^. Briefly, 8-day-old females starved for 24 hours were offered an artificial infectious blood meal in 3 rounds of 15 minutes using a Hemotek membrane-feeding apparatus with porcine intestine as membrane. Blood meals consisted of a 2:1 mix of washed erythrocytes (rabbit erythrocytes for DENV experiments and human erythrocytes for the YFV experiment) and virus suspension. Human erythrocytes were obtained in accordance with relevant guidelines and regulations through a protocol approved by the French Ethical Committee Ile-de-France I, as described above. Adenosine triphosphate (Merck) was added as a phagostimulant^37^ to the blood meal at a final concentration of 10 mM. In DENV experiments, mosquitoes were exposed to 1.16–1.38 ×10^7^ focus-forming units (FFUs)/mL of DENV-1. In the YFV experiment, they were exposed to 1.84 ×10^6^ FFUs/mL of YFV. Fully engorged females were sorted on wet ice, transferred into 1-pint cardboard containers and maintained in a climatic chamber (28 °C, 70% relative humidity, 12:12 hour light cycle) with permanent access to 10% sucrose solution. Fourteen days after the blood meal, mosquitoes were cold-anesthetized to remove wings and legs for DENV experiments or paralyzed with triethylamine for the YFV experiment^12^ to collect saliva samples *in vitro*^38^. The proboscis of each female was inserted into a 20-µL pipet tip containing 5 µL of fetal bovine serum (FBS). After 30 minutes of salivation, head and body were separated and stored individually at −80 °C. The saliva-containing FBS was mixed with 45 µL of Leibovitz’s L-15 medium and immediately inoculated onto sub-confluent C6/36 cells for titration by FFA, as described above, without subsequent dilution.

### Virus Detection

In DENV-1 experiments, bodies were homogenized individually in 300 µL of PBS. Body homogenates were centrifuged and viral RNA was extracted using the NucleoSpin RNA Virus kit (Macherey-Nagel) according to the manufacturer’s instructions. Detection of DENV-1 RNA was performed with the EXPRESS one-step Superscript qRT-PCR kit (ThermoFisher Scientific) using the following program: 30 min at 45 °C, 2 min at 95 °C, 55 cycles of 15 sec at 95 °C and 30 sec at 60 °C with a final step of 2 min at 25 °C. The 25-µL reaction volume contained 1x of reaction mix, 1.4 µM of primers, 5 µM of probe (forward: 5′-AAGGACTAGAGGTTAKAGGAGACCC-3′; reverse: 5′- CGWTCTGTGCCTGGAWTGATG-3′; probe: 5′-TCTGGTCTTTCCCAGCGTCAATATGCTGTT-3′)^39^ and 5 µL of RNA extract. In the YFV experiment, viral RNA was extracted by grinding bodies individually in 300 µL of squash buffer (Tris 10 mM, NaCl 50 mM, EDTA 1.27 mM with final pH adjusted to 8.2) supplemented with proteinase K (1 µL for 55.5 µL of squash buffer) and by incubating 100 µL of body homogenates for 5 min at 56 °C followed by 10 min at 98 °C. Detection of YFV RNA was performed using a two-step RT-PCR reaction to generate a 192-bp amplicon located in a conserved region of the *NS3* gene of YFV. Total RNA was reverse transcribed into cDNA with random hexamers using M-MLV reverse transcriptase (ThermoFisher Scientific) using the following program: 10 min at 25 °C, 50 min at 37 °C and 15 min at 70 °C. The cDNA was subsequently amplified using DreamTaq DNA polymerase (ThermoFisher Scientific). The 20-µL reaction volume contained 1x of reaction mix and 10 µM of primers (forward: 5′-GCGTAAGGCTGGAAAGAGTG-3′; reverse: 5′-CTTCCTCCCTTCATCCACAA-3′)^40^. The thermocycling program was 2 min at 95 °C, 35 cycles of 30 sec at 95 °C, 30 sec at 60 °C, and 30 sec at 72 °C with a final extension step of 7 min at 72 °C. Amplicons were visualized by electrophoresis on a 2% agarose gel. In all experiments, mosquito heads were homogenized in 300 µL of Leibovitz’s L-15 medium supplemented with 2% FBS, centrifuged, and assayed by a qualitative version (without end-point dilution) of the FFA described above.

### Olfactometer bioassays

**Figure 4:**
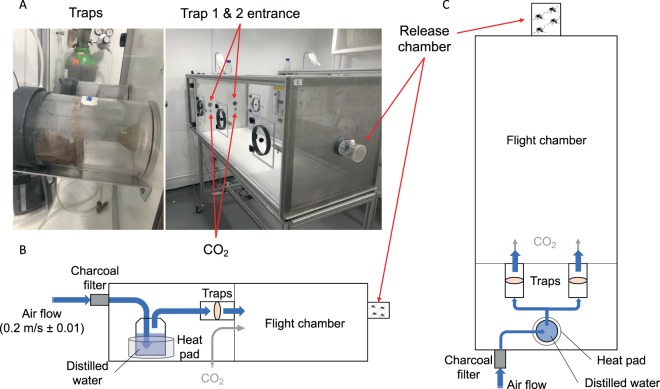
Dual-port olfactometer apparatus. (**A**) Pictures and (**B**,**C**) schematics (**B**: side view; **C**: top view) of the experimental setup to measure attraction to human odor.

Specific attraction of *Ae. malayensis* to human odor was evaluated using a dual-port olfactometer (160 ×60 ×43 cm) in a controlled environment room at the London School of Hygiene and Tropical Medicine (Fig. 4A). The experiments included *Ae. aegypti* as an anthropophilic control. To mimic human scent, a sheer polyamide stocking washed in 70% ethanol was worn by the experimenter for 12 hours and stored at −20 °C until use. An unworn stocking, which had been cleaned in the same manner, was used as control. Prior to the experiment, 5- to 7-day-old mosquitoes were deprived of sucrose for 24 hours. Batches of 20 to 30 females were transferred into a release chamber and allowed to acclimatize for 1 hour. Stockings were thawed during 1 hour and placed inside the traps according to two distinct experimental designs. The first design, denoted “CO_2_ + Human odor vs. CO_2_ only” hereafter, consisted of one trap with human odor and one trap with no odor. The second design, denoted “CO_2_ only vs. CO_2_ only” hereafter, consisted of both traps without human odor and was used as a negative control. This negative control ensures that the device is not intrinsically biased and that mosquitoes are not preferentially attracted to one of the traps in the presence of CO_2_ only, a known mosquito attractant. The air stream was heated and humidified, then subsequently directed to traverse either trap until it reached the release chamber. The air stream was supplemented with CO_2_ (5%) released at the entrance of each trap at a rate of 175 mL/min. The air speed at the exit of the traps was set at 0.2 m/sec with a variation of no more than 0.01 m/sec between each trap (Fig. 4B,C). The sliding door of the release chamber was opened, and mosquitoes were allowed to enter the flight chamber for 20 min. During each run, mosquitoes that flew upwind towards the odor were caught inside the traps. A total of 9 replicates were performed for each mosquito species to test their attraction to human odor (CO_2_ + Human odor vs. CO_2_ only), in addition to 6 replicate runs for each species to control for any bias in the olfactometer (CO_2_ only vs. CO_2_ only). We used a Latin square design to randomize the position of each trap between replicate runs by systematically switching traps between the right and left openings. Flight activity was measured as the percentage of mosquitoes that exited the release chamber after 20 min. Attraction to human odor was estimated as the percentage of trapped mosquitoes that chose the trap with human odor over the trap with no odor in the CO_2_ + Human odor vs. CO_2_ only design. Our measure of attraction to human odor ranged from 0% (full attraction to CO_2_ without human odor) to 100% (full attraction to CO_2_ with human odor) with 50% indicating a lack of either attraction.

### Statistical analyses

All statistical analyses were performed using the R software version 3.5.2^41^ and graphical representations were generated with the R package *ggplot2*^42^. Vector competence was evaluated using four conventional indices. Infection rate (IR) was estimated as the proportion of blood-fed females that became infected. Dissemination rate (DR) was estimated as the proportion of infected females that developed a systemic infection (i.e., with an infected head). Transmission rate (TR) was estimated as the proportion of females with a disseminated infection that released virus in their saliva. Transmission efficiency (TE) is a summary metric estimated as the overall proportion of blood-fed females with virus-positive saliva. Vector competence indices (IR, DR, TR and TE) were analyzed with a logistic regression model in which each individual mosquito was associated with a binary variable (infected = 1, uninfected = 0), followed by an analysis of deviance with the R package *car*^43^. For DENV experiments, the initial statistical model included the species, the experiment and their interaction as explanatory variables. The experiment effect between the two DENV experiments was statistically non-significant overall and subsequently removed from the model. In the olfactometer bioassays, flight activity and attraction to human odor were analyzed using a logistic regression model where each individual mosquito was associated with a binary variable (flight activity: active = 1, inactive = 0; attraction: trap 1 = 0, trap 2 = 1), followed by an analysis of deviance with the R package *car*^43^. Both analyses accounted for the replicate run effect, which was statistically non-significant overall. Multiple comparison between conditions was performed using the estimated marginal means followed by Tukey’s post-hoc test using the R package *emmeans*^44^.

## Data Availability

The datasets analyzed during the current study are available from the corresponding authors on reasonable request.
